# Retrospective Review of Outcomes in Non-Invasive Mucinous Appendiceal Neoplasms with and without Peritoneal Spread: A Cohort Study

**DOI:** 10.3390/curroncol29120714

**Published:** 2022-11-23

**Authors:** Arkadii Sipok, Jonathan M. Dort, Anthony Visioni, Lana Bijelic

**Affiliations:** 1 Department of Surgery, Inova Medical Campus, 3300 Gallows Rd, Falls Church, VA 22042, USA; 2 Department of General Surgery, Cleveland Clinic, Akron General Ave, Akron, OH 44307, USA; 3 Consorci Sanitari Integral, Hospital Sant Joan Despì, 90 Moises Broggi Calle Jacint Verdaguer, Sant Joan Despí, 08970 Barcelona, Spain

**Keywords:** pseudomyxoma peritonei, mucinous carcinoma peritonei, peritoneal metastases, cytoreductive surgery, heated intraperitoneal chemotherapy, appendix, LAMN, mucinous adenocarcinoma, appendiceal cancer, mucinous neoplasm

## Abstract

Patients treated surgically for local non-invasive mucinous appendiceal neoplasm (NI-MAN) may recur with the development of peritoneal dissemination (PD). The risk of recurrence and predictive factors are not well studied. Patients with NI-MAN, with or without peritoneal dissemination at presentation, were included. Patients with limited disease underwent surgical resection only. Patients with peritoneal dissemination underwent cytoreductive surgery (CRS) with or without hyperthermic intraperitoneal chemotherapy (HIPEC). Patients without PD (nPD) were compared to those who presented with PD. Thirty-nine patients were included, 25 in nPD and 14 in PD. LAMN was diagnosed in 96% and 93% of patients in nPD and PD, respectively. Acellular mucin on the peritoneal surface was seen in 16% of nPD patients vs. 50% of PD patients (*p* = 0.019). Two (8%) patients in the nPD group who had LAMN without wall rupture recurred, at 57 and 68 months, with a PCI of 9 and 22. The recurrence rate in the PD group was 36%. All recurred patients underwent CRS+HIPEC. A peritoneal recurrence is possible in NI-MANs confined to the appendix even with an intact wall at initial diagnosis. The peritoneal disease may occur with significant delay, which is longer than a conventional follow-up.

## 1. Introduction

Appendiceal neoplasms are rare, with an annual incidence of 0.15 to 0.8 per 100,000 [1], with non-invasive mucinous appendicular neoplasms (NI-MAN) being the most common epithelial neoplasms. The pathologic characteristics of these appendix-localized mucinous tumors include cell atypia, loss of muscularis mucosae, submucosal fibrosis, “pushing invasion”, dissection of acellular mucin in the wall, sinous or flattened epithetlial growth, rupture of the appendix, or mucin, and/or cells outside the appendix. Depending on the degree of cytological atypia, they can also be classified as low or high grade mucinous appendiceal neoplasms (LAMN/HAMN) [2,3].

Clinical manifestations of this tumor range from the incidental finding of mucocele treated with appendectomy to well-established disease with disseminated mucin throughout the peritoneal cavity resulting in pseudomyxoma peritonei (PMP), a progressive and fatal peritoneal surface malignancy [1,2] which requires treatment with cytoreductive surgery (CRS) in combination with hyperthermic intraperitoneal chemotherapy (HIPEC) [3,4,5,6].

Generally, a diagnosis of NI-MAN is associated with a good overall prognosis and survival, especially when confined to the appendix. Nevertheless, even without evidence of rupture of the appendix, these tumors are the most common precursors of PMP that can recur even after appropriate treatment [7,8].

The risk factors associated with disease recurrence and poor patient outcomes seen in some patients with NI-MAN are not well understood, with few reports currently available [9,10,11]. Patients who present with established peritoneal spread at the time of initial diagnosis appear to have the highest risk for recurrence [8,9,12]. On the other end of the spectrum, patients who present without or with minimal peritoneal disease appear to have a reduced risk of recurrence but the level of risk and the predictive factors associates with potential peritoneal progression are not well understood [12]. Consenquently, optimal management of patients with completely resected LAMN and HAMN, especially if rupture or mucin on the surface are seen is not clearly established with recommendations ranging from observation to prophylactic CRS and HIPEC [12]. Therefore, the aim of this study is to evaluate patient and primary tumor characteristics associated with peritoneal spread which may help define a subgroup of patients with resected LAMN or HAMN who are at higher risk of peritoneal recurrence.

## 2. Materials and Methods

### 2.1. Study Design and Settings

A retrospective cohort study was conducted to evaluate patient and tumor characteristic as well as recurrence rates of NI-MAN patients with localized disease confined to appendix and those with peritoneal dissemination of cellular or acellular mucin at presentation.

### 2.2. Population and Data Source

Patients with NI-MAN treated at a single institution between January 2015 to December 2018 were included. Data were obtained through medical chart review. Carr et al. classification was used for histopathology grading of appendiceal neoplasms [13]. Inclusion criteria were: (1) patients diagnosed with low- or high-grade mucinous neoplasm (LAMN/HAMN) of appendiceal primary (2) with or without low- or high-grade mucinous carcinoma peritonei (LGMCP/HGMCP) at presentation, with prior history of LAMN or HAMN, and treated surgically (3) patients with initial diagnosis at the study center, at an external practice with a referral to the study center, or previously had this diagnosis with treatment at external institutions and seeking further treatment at the study center. Patients < 18 years were excluded. The Institutional Review Board at the Inova Medical Campus approved the study.

### 2.3. Intervention

Patients with disease confined to the appendix underwent surgical treatment with appendectomy or right hemicolectomy (including outside institutions), those with peritoneal dissemination underwent either only CRS (outside institutions) or CRS and HIPEC (CRS+HIPEC) (in our institution) if tumor was found beyond the right lower quadrant (RLQ). In the cases of peritoneal recurrence, patients were treated with CRS+HIPEC. The CRS+HIPEC technique has been described elsewhere [14]. HIPEC was delivered in a closed fashion with mitomycin C (15 mg/m^2^) and doxorubicin (15 mg/m^2^) administered intraperitoneally for 90 min with targeted temperature of 41–42 °C, and with simultaneous intravenous infusion of 5-fluorourocil.

### 2.4. Follow-Up

Follow-up was at 1, 3, 6, 12, 18 months and annually thereafter, and included physical examination, serum levels of carcinoembryonic antigen (CEA), carbohydrate antigen (CA 19-9), and imaging every 6 months for at least 2 years. Disease recurrence was identified by imaging studies (CT-scan, PET-scan or MRI), elevated biochemical markers (CEA, or CA 19-9), and/or clinical presentation (i.e., bowel obstruction).

### 2.5. Variables and Scores

Patient and tumor data, as well as surgical characteristics, including age, gender, presenting symptoms, CEA, CA 19-9 and CA 125 at presentation, imaging findings, histological subtype, presence of appendix perforation, extent of peritoneal disease and surgery, were collected. Family history of malignancy referred to any oncology disease in a first degree relative. Tumor size was reported based on measurements in either the operative note or pathology report.

Appendices resected at our institution were submitted to pathology entirely and thoroughly examined for presense of gross rupture or microperforation. Histopathologic slides were requested from other institutions and reviewed by an expert pathologist. Rupture of the appendiceal wall was defined as evidence of wall rupture with complete penetration of intraluminal mucin into the serous surface of the appendix and on the basis of a the pathology report [15]. Tumor burden extent was assessed with the peritoneal carcinomatosis index (PCI) described by Jacquet et al. [16]. Completeness of cytoreduction (CC) was recorded with CC score, where CC-0 - no visible tumor remained within the abdomen, CC-1—residual tumor nodules <2.5 mm, CC-2—residual tumor nodules ≥2.5 mm, and CC-3—residual tumor ≥2.5 cm [16].

### 2.6. Study Outcomes

The primary outcome was the rate of peritoneal recurrence. Recurrence free survival was defined as the time from definitive treatment and the date of the last contact (censored), or date of recurrence (event) or death, whichever occurred first.

### 2.7. Statistical Analysis

Patients with no peritoneal dissemination (nPD) who had LAMN/HAMN limited to appendix with or without mucin over appendix and with or without mucin present on RLQ peritoneum at the site of direct contact with appendix were compared to patients with peritoneal dissemination (PD) of mucin from LAMN/HAMN found outside of RLQ. The statistical analysis was performed with the use of SPSS statistical software (IBM, v.26, Armonk, NY, USA). Patient characteristics were summarized and compared between the two cohorts using non-parametric tests. Continuous variables are presented as medians with interquartile range (IQR), and categorical variables as frequencies and proportions (%). Fisher’s exact test and Wilcoxon rank-sum test were used for categorical and continuous variables, respectively. Statistical significance was defined at 0.05 level, with statistical tests analyzed as two-sided.

## 3. Results

### 3.1. Participants

Out of 188 appendiceal tumor patients, 39 NI-MAN patients were identified. Twenty-five (64%) patients had local LAMN/HAMN at the time of initial presentation (nPD) and 14 (36%) had peritoneal dissemination (PD).

### 3.2. Patient Characteristics and Disease Presentation

Table 1 reflects characteristics of the study cohorts. The median age did not significantly differ between nPD and PD patients, 59 (IQR: 52–63) vs. 64 (IQR: 59–69), respectively, (*p* = 0.098). There were 60% females in nPD group and 71% in PC (*p* = 0.729).

At the time of initial presentation, the most common symptom in both cohorts was abdominal pain or discomfort, 48% in nPD and 50% in PD group (*p* > 0.999). Symptoms of appendicitis were observed in 8 (32%) nPD patients, while PD patients presented with abdominal fullness (43%), fatigue (14%), and weight loss (7%). Serum tumor markers were obtained preoperatively only in patients with peritoneal spread of the disease, with a median CEA 6.2 (IQR: 2.2–11.4), median CA-19.9 26 (IQR: 11.7–37.2), and median CA-125 85 (IQR: 31.6–93.0). Imaging studies identified an enlarged appendix in 60% of nPD and 14% of PD patients (*p* = 0.022). Ascites (64%), peritoneal involvement (57%) and omental caking (29%) were seen only in PD patients.

### 3.3. Surgical and Pathology Outcomes

All patients in the nPD group underwent local resection: 15 (60%) had appendectomy, 6 (24%) had right hemicolectomy and 4 (16%) had cecectomy. The minimally invasive approach was used in 22 (88%) of nPD and 3 (21%) of PD patients, with conversion to open at rates of 16% and 7%, respectively (*p* < 0.001). CRS+HIPEC was performed in 7 (50%) of PD patients as initial treatment. Median PCI in these patients was 23.5 (IQR: 22–25). Complete cytoreduction (CC 0/1) was achieved in all CRS+HIPEC cases. Other PD patients were treated with CRS only.

Pathology review showed no appendiceal rupture in 20 (80%) nPD patients; however, 2 (8%) of these patients had acellular mucin over the appendix and 3 (12%) patients had a small amount of mucin in the RLQ. Microscopic examination of surgical margins showed negative surgical margins (R0) in 24 (96%) nPD patients. HAMN was identified in 1 (4%) and 1 (8%) of nPD and PD patients, respectively (*p* = 1.00). Acellular extra-appendiceal mucin was found in 5 (20%) vs. 14 (100%), while cellular mucin was found only in 7 (50%) PD patients (*p* = 0.001). LGMCP was diagnosed in 6 (43%) and HGMCP in 1 (7%) of PD patients. After initial surgery, 1 (4%) nPD patient with HAMN received adjuvant systemic chemotherapy (Table 2).

### 3.4. Recurrence Rate

Median follow-up was 25 months (confidence interval [CI] 95%, 22–78). No patients were lost for follow-up. Two (8%) nPD patients and 5 (36%) PD patients recurred (*p* = 0.075). All patients with recurrence underwent CRS+HIPEC with no recurrence registered after that. The small number of events prevented further analysis of potential prognostic factors. One nPD patient recurred 57 months after appendectomy and had elevated CEA (23 ng/mL). In this case, the appendix specimen from the primary surgery was reviewed and LAMN without gross or micro perforation, extra-appendiceal mucin or cells was confirmed. At subsequent CRS+HIPEC, PCI was 9 and CC-0 was achieved. Peritonectomies were performed in the pelvis, anterior abdominal wall and omental bursa; greater omentectomy, hysterectomy with bilateral adnexectomy and excision of tumor from the retro-hepatic space were carried out. Another nPD patient recurred in 68 months after appendectomy with signs of tumor throughout the abdominal cavity on CT scans, normal level of CEA and elevated CA 19-9. Review of pathology from the primary surgery showed LAMN without macro or micro perforation of the appendix and no mucin or cells outside the appendix. At the time of CRS+HIPEC, PCI was 22 and CC-1 was accomplished. Individual characteristics of recurred cases are in Table 3.

## 4. Discussion

In this study, we report the recurrence rate in NI-MAN patients with local disease and those with peritoneal dissemination of cellular or acellular mucin. Whereas the rate of peritoneal recurrence was expectedly higher in the PD group, two recurrences in the nPD group in patients with no intraoperative and pathological signs of appendiceal wall integrity disruption were surprising findings. Interestingly, the recurrence in both these cases occurred over a long period of time, at 57 and 68 months after surgery, which is beyond the scope of standard follow-up of local tumors of the appendix. These data indicate that peritoneal recurrence is possible even in tumors confined to the appendix without signs of rupture and may occur after a significant period of time.

It has been widely accepted that if the appendix is removed with intact integrity of the wall during the surgical treatment of local NI-MAN, the risk of peritoneal recurrence is low, if it exists at all [17,18,19,20]. Several studies showed that peritoneal recurrence after local non-ruptured NI-MAN is extremely rare. Misdraji et al. reported outcomes of 27 LAMN patients after appendectomy and found no PD recurrence during a 6-year follow-up [21]. Another study of 17 local LAMN patients with or without extra-appendiceal mucin also showed no peritoneal recurrence at a median follow-up of 50 months [22]. Similar results were shown by Pai et al. who observed only 1 recurrence in a group of 14 LAMN patients with acellular extra-appendiceal mucin [9]. The rare frequency of recurrence in patients with local NI-MAN after surgical treatment is confirmed by findings in our study that showed recurrence only in two (8%) nPD patients. However, in contrast with data from previous studies, both recurrences were diagnosed in patients with local NI-MAN without signs of a ruptured appendix. This raises concern that intraperitoneal spread of NI-MAN is possible even without compromising appendiceal wall integrity.

There is no consensus on the follow-up schedule and duration after resection of a mucinous neoplasm due to the paucity of data regarding the progression of this rare disease, especially with isolated LAMN/HAMN, and is based on the experience of individual centers, and on the fact that most relapses occur within 5 years after surgery. Current guidelines do not support long-term follow-up if the appendix is resected without rupture of the wall or mucin entering the peritoneum, but recommends observation with CT/MRI and tumor markers for 5 years if the peritoneum has been affected [5]. Foster et al. [22] evaluated patients with serum tumor markers and CT before surgery, repeated every 4–6 months after surgery, followed by diagnostic laparoscopy if radiological abnormalities were found. In addition, laparoscopic observation was carried out regardless of the results of imaging examination. Guaglio et al. [11] performed physical examination, CT and serum tumor markers 3 months after the initial assessment, then every 6 months for the first 5 years and every year thereafter. They identified 1 recurrence 18 months after isolated LAMN resection. Our data support the need for appropriate follow-up even for patients after isolated LAMN resection. One patient who had a relapse 68 months after surgery had an advanced disease with PCI 22 at the time of relapse, which could have been recognized earlier during routine follow-up. In another patient, PD developed 57 months after index surgery, close to the recommended 5-year follow-up. Table 4 summarizes the current literature on LAMN/HAMN and recurrence rate. Given the low risk and long time to disease progression in patients with isolated LAMN, the question is whether the current surveillance approach is appropriate while PMP risk factors are still being studied. Our data suggest that a less intense but sufficiently long follow up period may be appropriate, such as physical examination, measurement of serum CEA and CA-19-9 tumor markers and CT scan or MRI every 6 months for the first 2 years and yearly after until at least 5 years. Because in patients with appendix perforation all recurrences occurred within 2 years after index surgery we suggest more intense surveillance especially during the first 2 years, including physical examination, measurement of serum CEA and CA-19-9 tumor markers and CT scan or MRI at 1, 3, 6, 12 and 18 months, and every 6 months thereafter for at least 5 years.

The development of PMP from a mucinous neoplasm of the appendix is a poorly understood process. Although not all patients with NI-MAN rupture progress to PMP, macroscopic or microscopic dissemination of mucin to the peritoneum increases the risk of PMP recurrence. In a study by Sueda et al. [24] in 136 patients with NI-MAN, tumor rupture on presentation was associated with the development of PMP. With the exception of patients with established peritoneal disease at the time of index surgery, 14% of patients with ruptured appendix subsequently developed PMP. Solomon et al. [8] in a study of 156 NI-MAN patients with PD, found that preoperative PCI > 12 was associated with disease recurrence. Yantiss et al. [12] in a study of 50 patients reported relapse in 2 (4%) patients with extra-appendiceal acellular mucin and 5 (33%) with extra-appendiceal neoplastic cells. The higher recurrence rate of PMP in patients with a ruptured appendix or high PCI is also supported by this study; 36% of relapsed patients had a ruptured appendix and mucin with neoplastic cells on the surface of the peritoneum during primary surgery. However, in 2 nPD patients who recurred, there was neither gross rupture nor microscopic evidence of invasion through the wall of the appendix. This could be explained by missed rupture of the appendix, therefore, we thoroughly reviewed operative notes and gross pathology reports, and in addition to that microscopic slides were reviewed with a pathologist. However, no rupture or micro perforation, or mucin on or outside appendix was found. Similar finding was reported by Guaglio et al. [11] where 1 patient recurred in 18 months with similar pathology. This raises concerns that other factors may contribute to the relapse of the disease.

There are several limitations of this study related to the retrospective design of the study and rarity of the disease: (1) small sample size, which resulted in inability to perform Cox regression analysis due to a low number of events, which is due to the good prognosis of the disease; (2) heterogeneity of patients within groups who received different treatment at initial presentation within each cohort; (3) missed data for some variables and no imputation of missed data (tumor markers, PCI score, etc.). (4) short follow-up for tumors with such good prognosis. While prospective studies are needed to overcome these deficiencies, we believe that our study may add more data to the existing pool.

## 5. Conclusions

A peritoneal recurrence is possible in NI-MANs confined to the appendix, even in those presenting without appendiceal rapture at the time of initial diagnosis. Moreover, in this case, a peritoneal disease may develop with significant delay, over 5 years after diagnosis of a primary tumor, which is longer than a conventional follow-up. More data are needed to identify risk factors of peritoneal dissemination other than an appendiceal rupture, and the development of a personalized follow-up schedule for patients possessing them is necessary.

## Figures and Tables

**Table 1:** Baseline and preoperative characteristics of study cohorts at the time of initial presentation.

Cohorts	No PD, N (%) N = 25 (64%)	PD, N (%) N = 14 (36%)	*p*-Value
Age, median (IQR)	59 (52–63)	64 (58.8–69.4)	0.098
Female	15 (60%)	10 (71.4%)	0.729
Family history of malignancy	9 (36%)	8 (57.1%)	0.314
Presenting Symptoms			
Abdominal pain/discomfort	12 (48%)	7 (50%)	>0.999
Sense of abdominal fullness/distension	0 (0%)	6 (42.9%)	0.001
Appendicitis	8 (32%)	0 (0%)	0.034
Fatigue	0 (0%)	2 (14.3%)	0.115
Weight loss	0 (0%)	1 (7.1%)	0.429
Other	3 (12%)	3 (21.4%)	0.775
Imaging findings			
Enlarged appendix	15 (60%)	2 (14.3%)	0.022
Simple appendicitis	2 (8%)	0 (0%)	0.658
Ascites	0 (0%)	9 (64.3%)	<0.001
Peritoneal carcinomatosis	0 (0%)	8 (57.1%)	<0.001
Omental caking	0 (0%)	4 (28.6%)	0.013
Mass in the pelvis/abdomen	4 (16%)	4 (28.6%)	0.652

PD: peritoneal dissemination; IQR: interquartile range.

**Table 2:** Surgical and treatment characteristics at the time of primary presentation.

Cohorts	No PD, N (%) N = 25 (64%)	PD, N (%) N = 14 (36%)	*p*-Value
Surgical approach			<0.001
Minimally invasive	18 (72%)	2 (14.3)	
Open	3 (12%)	11 (78.6)	
Converted to open	4 (16%)	1 (7.1)	
PCI, median (IQR)	N/A	23.5 (22-25)	N/A
CC 0–1, n (%)	N/A	7 (50)	N/A
Surgical margins			N/A
R0	24 (96)	N/A	
R1	1 (4)	N/A	
Histopathologic subtype of primary tumor			>0.999
LAMN	24 (96)	13 (92.9)	
HAMN	1 (4)	1 (7.1)	
Periappendiceal mucin			<0.001
None	20 (80)	0 (0)	
Present	5 (20)	14 (100)	
Peritoneal disease			<0.001
Acellular mucin	3 (16)	7 (50)	
LGMCP	0 (0)	6 (42.9)	
HGMCP	0 (0)	1 (7.1)	
Adjuvant chemotherapy	1 (4)	0 (0)	>0.999

PD: peritoneal dissemination; IQR: interquartile range; PCI: peritoneal carcinomatosis index; CC: completeness of cytoreduction score; R0: negative margins; R1: microscopic positive margins; LAMN: low-grade appendiceal mucinous neoplasm; HAMN: high-grade appendiceal mucinous neoplasm; LGMCP: low-grade mucinous carcinoma peritonei; HGMCP: high-grade mucinous carcinoma peritonei.

**Table 3:** Clinicopathologic characteristics of recurred patients at the time of index surgery.

PatientID	Age	Sex	Initial Treatment	Primary Tumor	Tumor Size (mm)	Peritoneal Disease	Surgical Margins	Time to Recurrence (Months)
nPD-1	66	F	Laparoscopic appendectomy	LAMN	70	No	Negative for tumor	57
nPD-2	62	F	Laparoscopic appendectomy	LAMN	30	No	Negative for tumor	68
PD-1	57	M	CRS+HIPEC (PCI: 24; CC-1)	LAMN	38	LGMCP	N/A	7
PD-2	58	F	Laparoscopic appendectomy and unilateral oophorectomy	HAMN	7	HGMCP	N/A	4
PD-3	65	M	CRS+HIPEC (PCI: 25; CC-1)	LAMN	10	LGMCP	N/A	13
PD-4	62	F	CRS+HIPEC (PCI: 21; CC-1)	LAMN	50	LGMCP	N/A	19
PD-5	61	F	Open appendectomy, hysterectomy with BSO	LAMN	Unknown	LGMCP	N/A	4

nPD: no peritoneal dissemination; PD: peritoneal dissemination; LAMN: low-grade appendiceal mucinous neoplasm; HAMN: high-grade appendiceal mucinous neoplasm; LGMCP: low-grade mucinous carcinoma peritonei; HGMCP: high-grade mucinous carcinoma peritonei; CRS+HIPEC: cytoreductive surgery and hyperthermic intraperitoneal chemotherapy; PCI: peritoneal carcinomatosis index; CC: completeness of cytoreduction score; BSO: bilateral salpingo-oophorectomy.

**Table 4:** Overview of previously published studies on LAMN/HAMN and their outcomes.

Authors (Year of Publication)	N	Appendiceal Primary Pathology	Extraappendiceal Dissemination at Presentation	Median Follow-Up (Months)	Recurrence Rate	Time to Recurrence
Misdraji et al. (2003) [21]	61	LAMN	Tumors confound to the appendix (n = 27)	72	0	-
			Acellular mucin involving avaries and tubes (n = 7)	18	2 (29%)	Not reported
			Low-grade mucinous neoplasms involving the peritoneum (n = 27)	72	18 (67%)	Not reported
Pai et al. (2009) [9]	57	LAMN	LAMN confound to appendix (n = 16)	59	0	-
			Acellular extraappendiceal mucin (n = 14)	48	1 (7%)	45 months
			LAMN with extraappendiceal neoplastic epithelium (n = 27)	53	21 (78%)	
Yantis et al. (2009) [12]	65	LAMN (n = 54) HAMN (n = 11) All had periappendiceal mucin	Acellular mucin (n = 50)	36	2 (4%)	(1) 56 months (2) 92 months
			Mucin with neoplastic epithelium (n = 15)		5 (33%)	Range: 24–87 months
Foster et al. (2016) [22]	23	LAMN	LAMN confound to appendix (n = 17)	50	0	-
			LAMN with peritoneal metastases (n = 5)	50	1 (20%)	24 months
Guaglio et al. (2018) [11]	41	LAMN	Acellular mucin outside appendix (n = 19) including: - Cellular mucin outside appendix (n = 3) - - Infiltration of near organs by tumor primary (n = 2)	58	2 (4.9%)	(1) 18 months (2) 22 months
Li et al. (2018) [23]	50	LAMN	PMP (n = 13)	53	0	-
Solomon et al. (2020) [8]	156		Acellular mucin (n = 25)	45	2 (8%)	Not reported
			Mucin with neoplastic epithelium (n = 131)		11 (8.4%)	Not reported
Sueda et al. (2020) [24]	138	LAMN HAMN	Ruptured appendix (n = 36) including: - gross mucin spillage (n = 16) - PMP (n = 8)	61	12 (8.7%)	5–20

LAMN: low-grade appendiceal mucinous neoplasm; HAMN: high-grade appendiceal mucinous neoplasm; PMP: pseudomyxoma peritonei.

## Data Availability

The data presented in this study are available on request from the corresponding author.
